# Supplementary material for: Developmental wave of programmed ganglion cell death in human retinal organoids
Source: bioRxiv. 2025 Jul 30:2025.07.25.666895. Preprint. [Version 1] doi: 10.1101/2025.07.25.666895 (PMC12324330; doi:10.1101/2025.07.25.666895)
Supplement: Supplement 1 [file NIHPP2025.07.25.666895v1-supplement-1.pdf]

bioRxiv preprint doi: https://doi.org/10.1101/2025.07.25.666895; this version posted July 30, 2025. The copyright holder for this preprint (which was not certified by peer review) is the author/funder, who has granted bioRxiv a license to display the preprint in perpetuity. It is made available under aCC-BY 4.0 International license.

Figure S1. Longitudinal quantification of fluorescent reporters in live retinal organoids. a) Fluorescence micrographs of whole-mount live transgenic organoids at 10 weeks of differentiation show expression of *VSX2*-Cerulean (retinal progenitors), *POU4F2*-eGFP (RGCs), and *RCVRN*-mCherry (photoreceptor precursors). b) Quantification of reporter expression normalized with VSX2-Cerulean. Bar graph represents mean ± SEM; individual samples are plotted. *$p<0.05$; **$p<0.01$. Scale bar: 100 µm.

Figure S2. Developmental wave of cell death primarily affects RGCs. Fluorescence micrographs of RO cryosections at 8 weeks of differentiation. a) Immunofluorescent staining for HuC/D (red) labels RGCs. b) Immunofluorescent staining for cleaved caspase3 (ClC3; green) labels cells undergoing apoptosis. c) Merged image showing co-localization of these markers. Scale bar: 100 µm.

bioRxiv preprint doi: https://doi.org/10.1101/2025.07.25.666895; this version posted July 30, 2025. The copyright holder for this preprint (which was not certified by peer review) is the author/funder, who has granted bioRxiv a license to display the preprint in perpetuity. It is made available under aCC-BY 4.0 International license.

Supplementary table 1. Antibodies for Western blot analysis

**Antibody**	**Source**	**Dilution**
E7 (Anti-β Tubulin)	Developmental Studies Hybridoma Bank	1:3000
β-actin (C4)	Santa Cruz Biotechnology (cat# sc-47778)	1:5000
Anti-BAX	Proteintech (cat# 50599-2-Ig)	1:12000
Anti-BCL2	Proteintech (cat# 60178-1-Ig)	1:2500
Anti-Caspase 3	Cell Signaling (cat# 9662s)	1:1500
Anti-Caspase 8	Proteintech (cat# 66093-1-Ig)	1:5000
Anti-Cleaved Caspase 9	Cell Signaling (cat# 7237P)	1:1000
Peroxidase Labeled Anti-Rabbit IgG (H+L)	Vector (cat# PI-1000)	1:3000
Peroxidase Labeled Anti-Mouse IgG (H+L)	Vector (cat# PI-2000)	1:3000

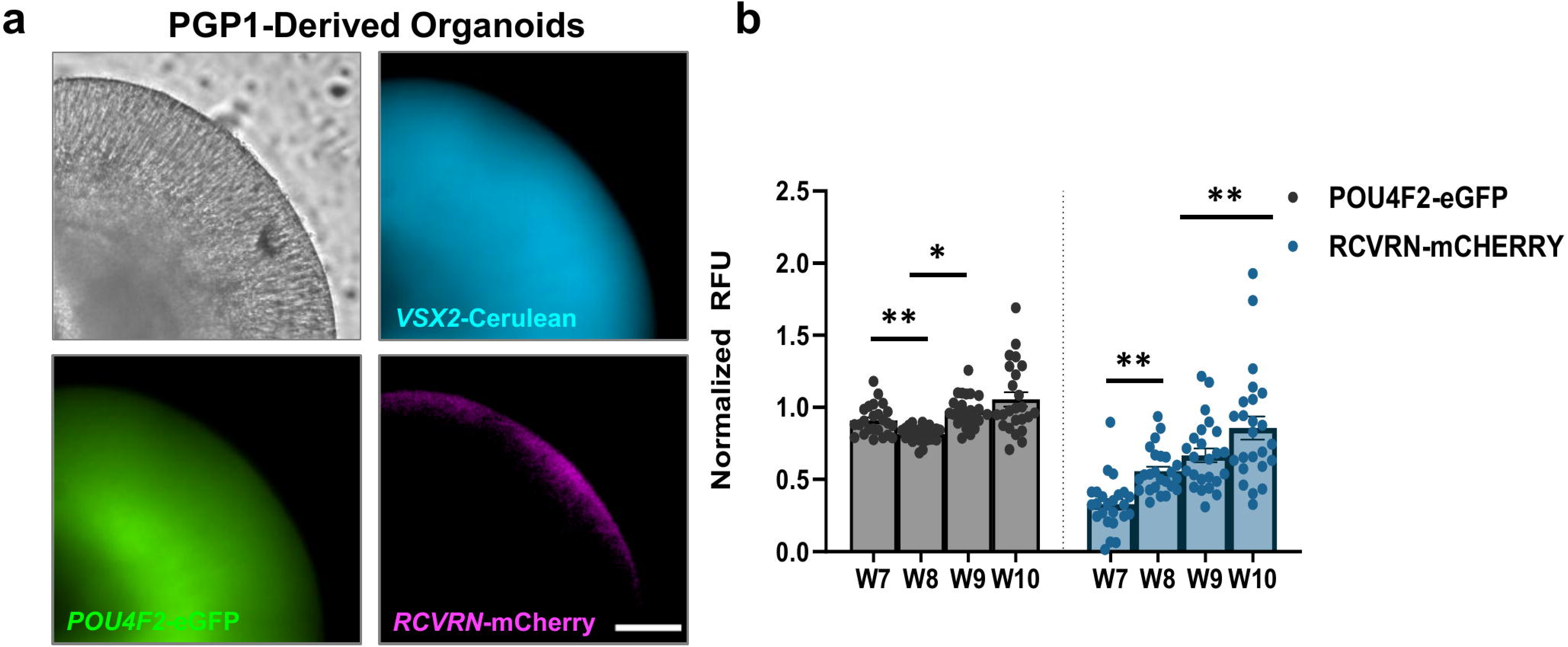

a
PGP1-Derived Organoids
VSX2-Cerulean
POU4F2-eGFP
RCVRN-mCherry
b
Normalized RFU
2.5
2.0
1.5
1.0
0.5
0.0
**
*
**
**
W7 W8 W9 W10
W7 W8 W9 W10
POU4F2-eGFP
RCVRN-mCHERRY

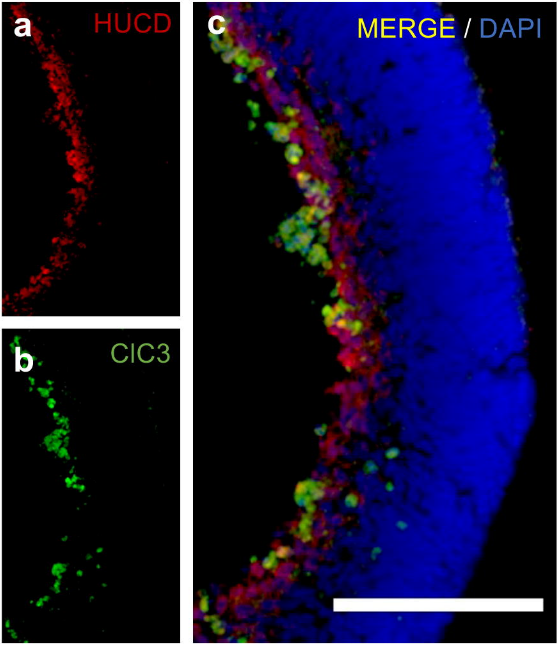
a
HUCD
b
ClC3
c
MERGE / DAPI
